# Post-CAR T-cell thyroid dysfunction: destructive thyroiditis identified through thyroid function monitoring in a single-center cohort

**DOI:** 10.3389/fonc.2026.1876913

**Published:** 2026-08-06

**Authors:** Yoshiyuki Fujita, Kyoko Yoshihara, Nobuto Utsunomiya, Fuyuki Yamagata, Tomoko Kumamoto, Saki Takahashi, Mami Samori, Atsushi Katayama, Masahiro Teramoto, Takayuki Inoue, Katsuji Kaida, Tazuko Tokugawa, Akihiro Sawada, Satoshi Higasa, Hiroya Tamaki, Satoshi Yoshihara

**Affiliations:** 1 Department of Hematology, Hyogo Medical University School of Medicine, Nishinomiya, Japan; 2 Department of Transfusion Medicine and Cellular Therapy, Hyogo Medical University Hospital, Nishinomiya, Japan

**Keywords:** CAR T-cell therapy, cytokine release syndrome, destructive thyroiditis, endocrine toxicity, thyroid dysfunction, thyroid function monitoring

## Abstract

**Background:**

Endocrine complications after chimeric antigen receptor (CAR) T-cell therapy remain poorly characterized. Thyroid dysfunction is well recognized during immune checkpoint inhibitor therapy, but thyroid function is not routinely incorporated into post-CAR T-cell toxicity assessment.

**Methods:**

After observing an index case of destructive thyroiditis after CAR T-cell therapy, scheduled thyroid function monitoring was implemented in the subsequent 94 consecutive CAR T-cell recipients at our institution. This single-center study therefore included the index case and 94 consecutive post-index patients treated with axicabtagene ciloleucel, lisocabtagene maraleucel, or idecabtagene vicleucel.

**Results:**

Two patients developed transient thyrotoxicosis compatible with destructive thyroiditis, corresponding to an observed proportion of 2 of 95 patients [2.1%; exact 95% confidence interval (CI), 0.3–7.4%]. Both cases occurred among the 12 patients who experienced grade ≥2 cytokine release syndrome (CRS; 2 of 12; exact 95% CI, 2.1–48.4%). One case was supported by markedly reduced uptake on thyroid scintigraphy, whereas the other was considered probable destructive thyroiditis. Minor or transient thyroid function abnormalities that did not meet the diagnostic criteria for destructive thyroiditis were also observed.

**Conclusions:**

Destructive thyroiditis may occur after CAR T-cell therapy and may be underrecognized because its manifestations overlap with other post-CAR T-cell inflammatory toxicities. The occurrence of both cases after grade ≥2 CRS represents a possible association and should be considered hypothesis-generating.

## Introduction

Chimeric antigen receptor (CAR) T-cell therapy has become an established treatment for relapsed or refractory B-cell malignancies and multiple myeloma. Its major acute toxicities include cytokine release syndrome (CRS) and immune effector cell-associated neurotoxicity syndrome (ICANS), both of which are closely related to systemic immune activation. Although the recognition and management of CRS and ICANS have improved substantially, less common immune-mediated complications after CAR T-cell therapy remain incompletely characterized.

Thyroid dysfunction is one of the most frequent endocrine immune-related adverse events associated with immune checkpoint inhibitors. Its reported frequency varies according to the agent, regimen, and definition used, but thyroid dysfunction is generally observed in approximately 5–20% of patients receiving immune checkpoint inhibitors and is more frequent with combination regimens (1, 2). Routine monitoring with thyroid-stimulating hormone (TSH) and free thyroxine (FT4) is recommended during immune checkpoint inhibitor therapy (3, 4). In contrast, thyroid dysfunction after CAR T-cell therapy has rarely been reported, and routine thyroid function monitoring is not generally incorporated into post-CAR T-cell toxicity assessment.

After observing an index case of destructive thyroiditis following CAR T-cell therapy, we implemented scheduled thyroid function monitoring in the subsequent consecutive patients undergoing CAR T-cell therapy at our institution. Here, we describe the observed frequency and clinical features of destructive thyroiditis in a single-center cohort comprising the index case and 94 consecutive post-index patients treated with commercially available CAR T-cell products, and discuss its potential relevance as an underrecognized endocrine toxicity after CAR T-cell therapy.

## Methods

**Figure 1:**
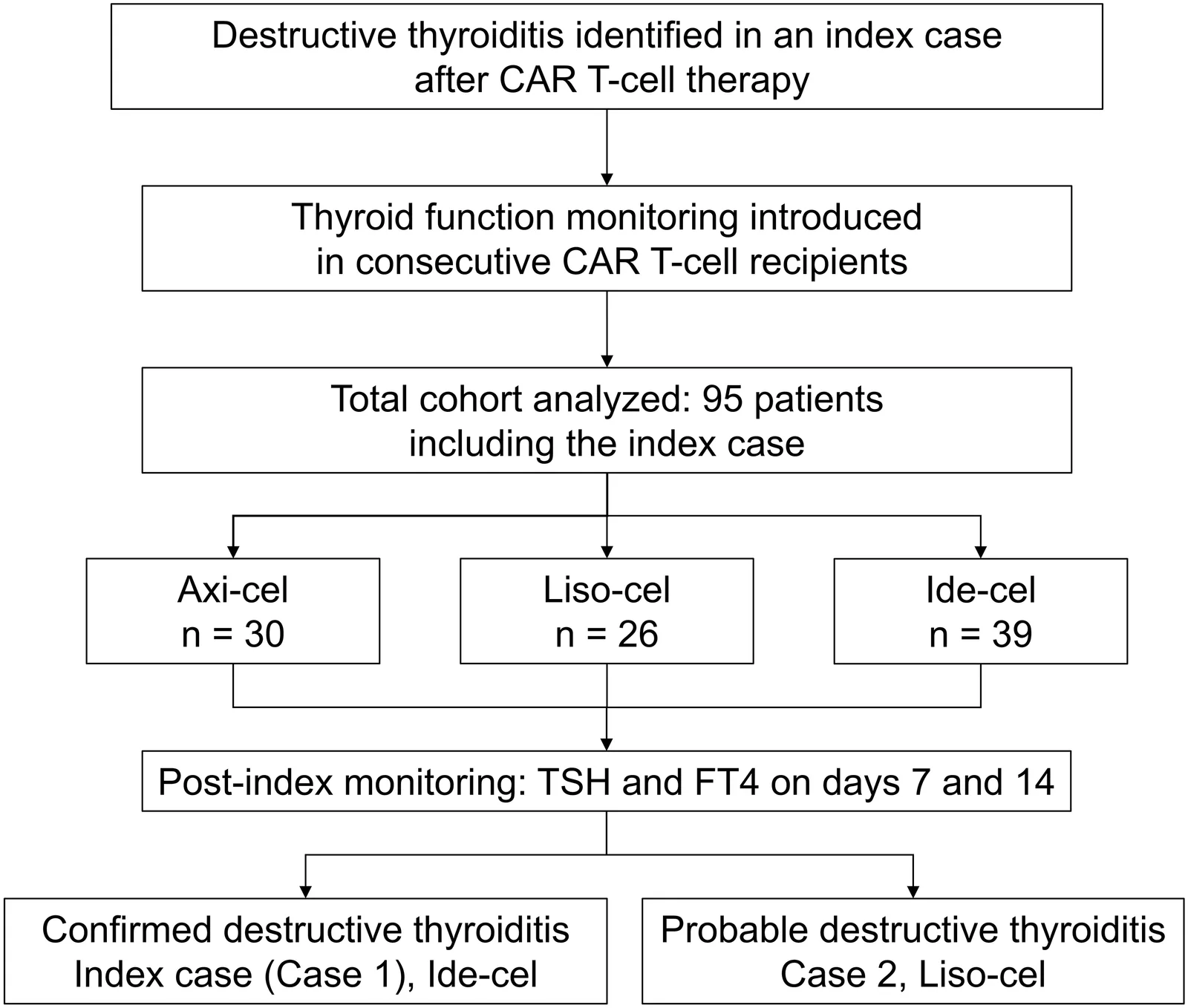
Study flow and thyroid function monitoring after CAR T-cell therapy. The cohort comprised an index case of destructive thyroiditis and 94 subsequent consecutive CAR T-cell recipients who underwent scheduled TSH and FT4 measurements on days 7 and 14. Destructive thyroiditis was identified in the index case after idecabtagene vicleucel and in one subsequent patient after lisocabtagene maraleucel.

This was a single-center observational cohort study conducted at Hyogo Medical University Hospital. After an index case of destructive thyroiditis following CAR T-cell therapy was identified, scheduled thyroid function monitoring was implemented in the subsequent 94 consecutive patients treated with CAR T-cell therapy. The present analysis therefore included the index case and 94 consecutive post-index patients, for a total of 95 patients treated between March 2019 and December 2025 (**Figure 1**).

Patients received one of the following commercially available CAR T-cell products: axicabtagene ciloleucel, lisocabtagene maraleucel, or idecabtagene vicleucel. Clinical data were collected regarding patient characteristics, underlying disease, CAR T-cell product, CRS and ICANS occurrence and severity, use of tocilizumab and corticosteroids, inflammatory markers, thyroid function, thyroid autoantibodies, imaging findings, and clinical course. Medical records were also reviewed for documented thyroid disease, thyroid-directed treatment, and available thyroid function results before CAR T-cell therapy.

In the index case, thyroid function was assessed when anorexia, fatigue, low-grade fever, and tachycardia developed after resolution of CRS and was subsequently monitored during follow-up. In the subsequent 94 consecutive patients, serum thyroid-stimulating hormone (TSH) and free thyroxine (FT4) levels were scheduled for measurement on days 7 and 14 after CAR T-cell infusion. Day 7 measurements were available in all 94 patients, and day 14 measurements were available in 93 of 94 patients. The institutional reference ranges were 0.50–5.00 μIU/mL for TSH and 0.90–1.70 ng/dL for FT4. Additional thyroid function testing, thyroid autoantibody measurement, thyroid ultrasonography, and thyroid scintigraphy were performed when clinically indicated. Destructive thyroiditis was diagnosed based on transient thyrotoxicosis characterized by suppressed TSH and elevated FT4, absence of serological evidence supporting Graves’ disease, a compatible clinical course, and imaging findings when available. Minor biochemical abnormalities that did not fulfill these criteria were recorded separately.

CRS and ICANS were graded according to the American Society for Transplantation and Cellular Therapy consensus criteria (5). This study was approved by the institutional review board of Hyogo Medical University Hospital and was conducted in accordance with institutional ethical standards. Written informed consent was obtained from all patients included in the study.

Observed proportions are presented as raw fractions and percentages. Two-sided 95% CIs were calculated using the exact binomial Clopper–Pearson method.

## Results

### Cohort characteristics

A total of 95 patients were analyzed, including 56 men and 39 women. Thirty patients received axicabtagene ciloleucel, 26 received lisocabtagene maraleucel, and 39 received idecabtagene vicleucel. Patients treated with CD19-directed CAR T-cell therapy had large B-cell lymphoma (LBCL) or follicular lymphoma, whereas those treated with BCMA-directed CAR T-cell therapy had multiple myeloma. Two patients were receiving levothyroxine replacement for pre-existing hypothyroidism or Hashimoto thyroiditis, and one patient had untreated chronic thyroiditis at the time of CAR T-cell infusion. None of these three patients developed destructive thyroiditis after CAR T-cell therapy.

CRS occurred in 28 of 30 patients treated with axicabtagene ciloleucel, 19 of 26 treated with lisocabtagene maraleucel, and 37 of 39 treated with idecabtagene vicleucel. ICANS occurred in 8, 6, and 1 patients, respectively. Tocilizumab and corticosteroids were frequently administered for CAR T-cell-related inflammatory toxicities (**Table 1**).

**Table 1:** Patient characteristics and CAR T-cell-related toxicities in the cohort.

Characteristic	Axi-cel (n=30)	Liso-cel (n=26)	Ide-cel (n=39)
Baseline characteristics			
Age, median (range), years	65.5 (45–76)	68.5 (54–78)	64 (53–76)
Male sex, n (%)	17 (56.7%)	15 (57.7%)	24 (61.5%)
Underlying disease			
LBCL	30 (100%)	21 (80.8%)	
FL	0	5 (19.2%)	
Multiple myeloma			39 (100%)
Number of prior lines			
1	17 (56.7%)	14 (53.8%)	—
2	9 (30.0%)	8 (30.8%)	8 (20.5%)
≥3	4 (13.3%)	4 (15.4%)	31 (79.5%)
Disease status at infusion			
PD	2 (6.7%)	3 (11.5%)	9 (23.1%)
SD	7 (23.3%)	1 (3.8%)	9 (23.1%)
PR/VGPR	11 (36.7%)	15 (57.7%)	5 (12.8%)
CR/sCR	10 (33.3%)	7 (26.9%)	16 (41.0%)
Triple-class refractory	—	—	24 (61.5%)
EMD+	—	—	19 (48.7%)
Toxicities after CAR-T infusion			
CRS incidence	28 (93.3%)	19 (73.1%)	37 (94.9%)
CRS onset, median days	2.5 (0–7)	1 (0–4)	1 (0–4)
CRS grade, n (1/2/3/4)	23/3/2/0	17/1/1/0	32/2/3/0
ICANS incidence	8 (26.7%)	6 (23.1%)	1 (2.6%)
ICANS onset, median days	7.5 (5–13)	7 (5–11)	3 (3–3)
ICANS grade, n (1/2/3/4)	3/1/3/1	1/4/1/0	1/0/0/0
Tocilizumab use	28 (93.3%)	19 (73.1%)	37 (94.9%)
Dexamethasone use	26 (86.7%)	16 (61.5%)	30 (76.9%)
Destructive thyroiditis	0	1 (3.8%)	1 (2.6%)

Axi-cel, axicabtagene ciloleucel; CAR, chimeric antigen receptor; CR, complete response; CRS, cytokine release syndrome; LBCL, large B-cell lymphoma; EMD, extramedullary disease; FL, follicular lymphoma; ICANS, immune effector cell-associated neurotoxicity syndrome; Ide-cel, idecabtagene vicleucel; Liso-cel, lisocabtagene maraleucel; PD, progressive disease; PR, partial response; sCR, stringent complete response; SD, stable disease; VGPR, very good partial response.

### Thyroid function monitoring and destructive thyroiditis

Among the 95 patients, two developed transient thyrotoxicosis compatible with destructive thyroiditis, corresponding to an observed proportion of 2 of 95 patients (2.1%; exact 95% CI, 0.3–7.4%). One case occurred after BCMA-directed CAR T-cell therapy with idecabtagene vicleucel, and the other occurred after CD19-directed CAR T-cell therapy with lisocabtagene maraleucel. Both patients were male, and both cases occurred among the 12 patients who experienced grade ≥2 CRS (2 of 12; exact 95% CI, 2.1–48.4%). Given the small number of events and the wide confidence interval, this finding was considered a possible association rather than evidence of a distinct high-risk subgroup. The clinical characteristics of the two patients are summarized in **Table 2**.

**Table 2:** Clinical characteristics of patients with destructive thyroiditis after CAR T-cell therapy.

Characteristic	Case 1	Case 2
Age/sex	67/Male	74/Male
Disease	IgA-κ multiple myeloma	Diffuse large B-cell lymphoma
CAR T-cell product	Ide-cel	Liso-cel
CRS	Grade 3	Grade 2
ICANS	None	Grade 3
Peak sIL-2R, U/mL	15,155	7,342
Peak ferritin, ng/mL	3,573	5,086
Symptoms at detection	Anorexia, fatigue, low-grade fever, tachycardia	None
Day of thyrotoxicosis detection	Day 18	Day 7
TSH at detection, μIU/mL	0.024	0.016
FT4 at detection, ng/dL	3.82	2.05
Peak FT4, ng/dL	7.77 on day 25	2.36 on day 14
TSAb	101%	105%
TRAb, IU/L	<0.5	<0.5
Anti-Tg antibody, IU/mL	<10.0	<10.0
Anti-TPO antibody, IU/mL	2.9	41.8
Thyroid ultrasonography	Mildly irregular margins; no increased vascularity; cystic lesions only	Homogeneous parenchyma with increased vascular signal
Thyroid scintigraphy	Markedly reduced uptake; uptake rate 0.3%	Not performed
Treatment for thyroiditis	No antithyroid therapy	No antithyroid therapy
Clinical course	Transient subclinical hypothyroidism on day 80; TSH normalized by day 109, while FT4 remained slightly below the reference range, without levothyroxine	FT4 normalized by day 55; no overt hypothyroidism during available follow-up
Interpretation	Destructive thyroiditis	Probable destructive thyroiditis

CRS, cytokine release syndrome; FT4, free thyroxine; ICANS, immune effector cell-associated neurotoxicity syndrome; Ide-cel, idecabtagene vicleucel; Liso-cel, lisocabtagene maraleucel; sIL-2R, soluble interleukin-2 receptor; Tg, thyroglobulin; TPO, thyroid peroxidase; TRAb, TSH receptor antibody; TSAb, thyroid-stimulating antibody; TSH, thyroid-stimulating hormone.

**Figure 2:**
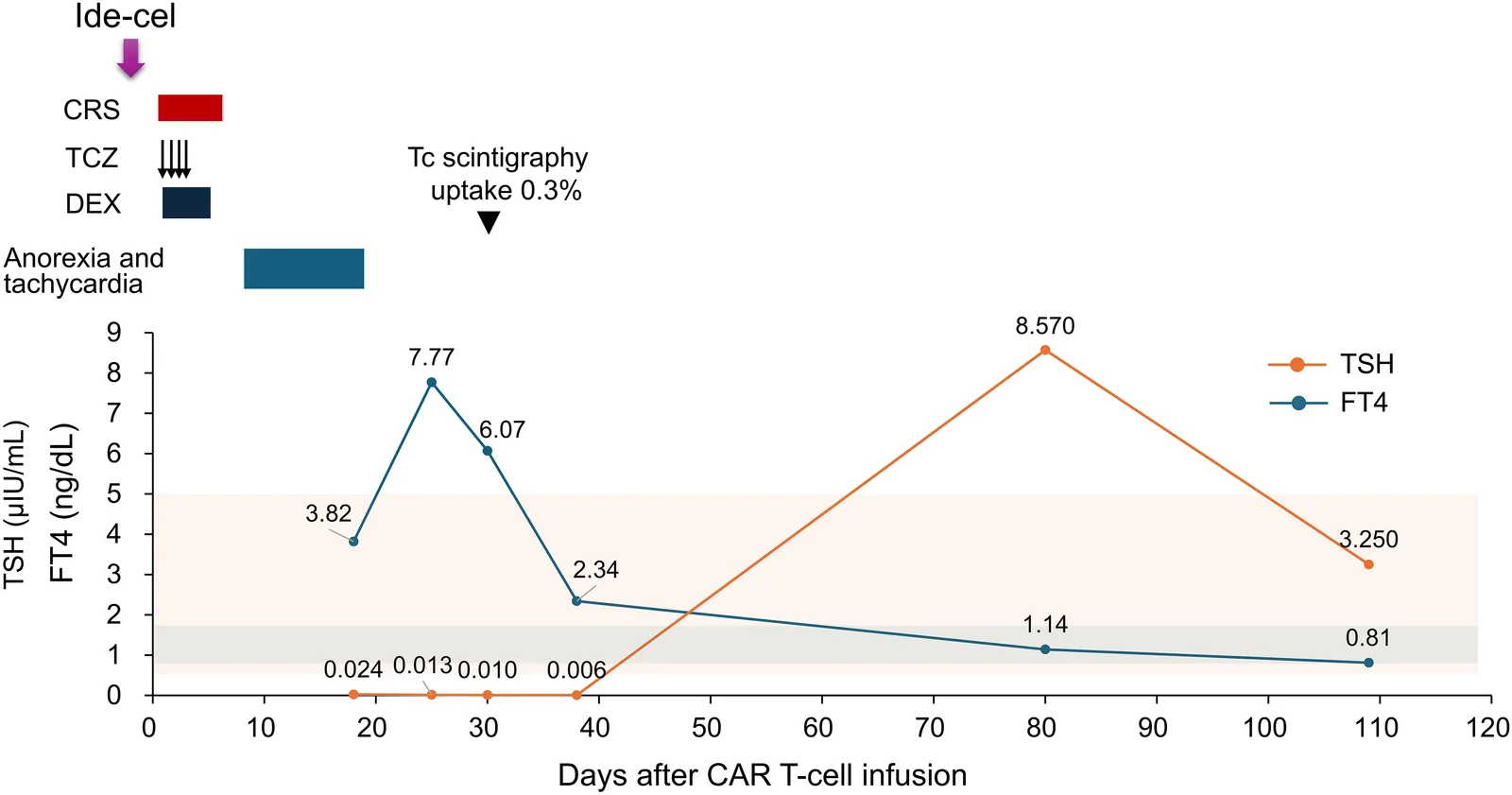
Clinical course of the index patient with destructive thyroiditis after idecabtagene vicleucel. After grade 3 CRS resolved, the patient developed anorexia, fatigue, low-grade fever, and tachycardia, followed by suppressed TSH and elevated FT4. FT4 peaked on day 25 and declined without antithyroid therapy, with a transient increase in TSH during recovery. Thyroid scintigraphy showed markedly reduced uptake of 0.3%. Shaded areas indicate the reference ranges.

The first patient was a 67-year-old man with relapsed or refractory IgA-κ multiple myeloma who received idecabtagene vicleucel. He developed grade 3 CRS, which resolved after treatment with tocilizumab and dexamethasone. Beginning on day 10, after resolution of CRS, he developed anorexia, fatigue, low-grade fever, and tachycardia (heart rate, 113 beats/min), which prompted thyroid function testing. On day 18, TSH was 0.024 μIU/mL and FT4 was 3.82 ng/dL. Follow-up testing on day 25 showed persistent thyrotoxicosis, with TSH 0.013 μIU/mL and FT4 7.77 ng/dL. Because the patient had IgA-κ multiple myeloma, paraprotein-related assay interference was considered. However, serum IgA decreased from 916 mg/dL on day −17 to 79 mg/dL on day 18 and 59 mg/dL on day 21, and serum free κ light chain was below the detection limit (<0.5 mg/L) on day 21. Total T4 was not measured. TSH receptor antibody was negative. Thyroid ultrasonography showed mildly irregular margins without increased vascularity, and technetium thyroid scintigraphy showed markedly reduced uptake, with an uptake rate of 0.3%. These findings supported the diagnosis of destructive thyroiditis. No antithyroid therapy was administered, and FT4 subsequently declined spontaneously. On day 80, TSH was transiently elevated to 8.57 μIU/mL with an FT4 level of 1.14 ng/dL, consistent with a transient subclinical hypothyroid phase. By day 109, TSH had normalized to 3.25 microIU/mL, while FT4 was slightly below the institutional reference range at 0.81 ng/dL, without levothyroxine therapy (**Figure 2**).

The second patient was a 74-year-old man with diffuse large B-cell lymphoma who received lisocabtagene maraleucel. He developed grade 2 CRS and grade 3 ICANS. Thyrotoxicosis was detected on day 7, with TSH 0.016 μIU/mL and FT4 2.05 ng/dL. On day 14, TSH was 0.089 μIU/mL and FT4 was 2.36 ng/dL. TSH receptor antibody was negative. Thyroid ultrasonography showed homogeneous thyroid parenchyma with increased vascular signal. Thyroid scintigraphy was not performed. FT4 subsequently normalized to 1.68 ng/dL by day 55 without antithyroid therapy. No overt hypothyroidism was documented during the available thyroid function follow-up. The transient course, suppressed TSH, negative TSH receptor antibody, and spontaneous improvement were considered compatible with probable destructive thyroiditis.

Among the remaining 93 patients, 57 had at least one TSH or FT4 value outside the institutional reference range on day 7 or day 14. The most common biochemical abnormality was low FT4, observed in 39 patients, either alone or with minor TSH changes. Isolated TSH suppression with FT4 remaining within the reference range was observed in 15 patients. The remaining abnormalities included mild TSH elevation or minimal FT4 elevation and did not fulfill the study diagnostic criteria for destructive thyroiditis. No additional patient was clinically diagnosed with destructive thyroiditis.

## Discussion

In this single-center cohort comprising an index case and 94 consecutive post-index patients, thyroid function assessment identified two cases of destructive thyroiditis among 95 CAR T-cell recipients. One case was supported by markedly reduced uptake on thyroid scintigraphy, and the other showed transient thyrotoxicosis with negative TSH receptor antibody and spontaneous improvement. These findings suggest that destructive thyroiditis may occur after CAR T-cell therapy and may be underrecognized in routine post-infusion care, while the small number of events precludes any conclusion regarding causality or incidence.

Thyroid dysfunction is well established as an endocrine immune-related adverse event during immune checkpoint inhibitor therapy. A systematic review and meta-analysis showed that endocrine dysfunction varies by immune checkpoint inhibitor regimen, with thyroid dysfunction being particularly associated with anti-PD-1/PD-L1-based therapy and more frequent with combination regimens (1). Because immune checkpoint inhibitor-associated thyroid dysfunction may initially be asymptomatic and can evolve from thyrotoxicosis to hypothyroidism, routine TSH and FT4 monitoring every 4–6 weeks is recommended during immune checkpoint inhibitor therapy (3, 4). By contrast, current CAR T-cell toxicity management primarily focuses on CRS and ICANS, and endocrine monitoring has not been standardized in this setting (5).

The mechanism of thyroid injury after CAR T-cell therapy remains unclear. Direct antigen-specific thyroid injury is unlikely, as thyroiditis occurred after both BCMA-directed and CD19-directed CAR T-cell therapy. However, CAR T-cell therapy can induce systemic immune activation and high-cytokine states, and inflammatory toxicities beyond direct target-specific tumor killing have been described after CAR T-cell therapy, including immune effector cell-associated hematotoxicity (6, 7). In the present cohort, both cases occurred among the 12 patients who experienced grade ≥2 CRS. This temporal pattern raises the possibility that systemic immune activation contributed to thyroid follicular injury, but only two events were observed, the exact 95% CI was wide, and chance association cannot be excluded.

Paraprotein-associated interference with thyroid hormone measurement is an important alternative explanation in patients with multiple myeloma. Monoclonal immunoglobulins may act as abnormal thyroid hormone-binding proteins or interfere analytically with thyroid hormone assays (9). We considered this possibility during the clinical course and repeatedly assessed the paraprotein burden. Serum IgA had already decreased from 916 mg/dL on day −17 to 79 mg/dL on day 18, when thyrotoxicosis was first documented, and to 59 mg/dL on day 21; serum free κ light chain was below the detection limit (<0.5 mg/L) on day 21. These findings made clinically meaningful paraprotein-related assay interference unlikely. Moreover, the FT4 elevation was accompanied by suppressed TSH, systemic symptoms and tachycardia, markedly reduced thyroid uptake of 0.3% on scintigraphy, spontaneous decline in FT4 without antithyroid therapy, and a transient subsequent increase in TSH during recovery.

Thyroiditis after CAR T-cell therapy may be difficult to recognize clinically. Tachycardia and nonspecific systemic symptoms such as fatigue, anorexia, low-grade fever, and malaise overlap with symptoms commonly observed during recovery from CRS, ICANS, infection, cytopenia, and systemic inflammation. In the index case, persistent tachycardia together with systemic symptoms prompted thyroid function testing, whereas the second case was detected through scheduled post-index monitoring. These observations suggest that thyroid dysfunction may be missed unless thyroid function is specifically assessed.

Minor biochemical thyroid function abnormalities were relatively common during early post-CAR T-cell monitoring. Among the remaining 93 patients, 57 had at least one TSH or FT4 value outside the institutional reference range on day 7 or day 14, most commonly reduced FT4 or isolated TSH suppression with FT4 remaining within the reference range. These findings suggest that mild thyroid function test abnormalities during the acute post-infusion period should be interpreted cautiously, as they may reflect non-thyroidal illness, glucocorticoid exposure, systemic inflammation, or pre-existing thyroid conditions rather than destructive thyroiditis. In contrast, persistent suppression of TSH with elevated FT4, compatible clinical findings, spontaneous evolution, and supportive imaging may help distinguish clinically relevant destructive thyroiditis from nonspecific biochemical fluctuations.

Only limited data are available regarding thyroid dysfunction after CAR T-cell therapy. Chen et al. reported two cases of Hashimoto thyroiditis after CD19-directed CAR T-cell therapy in patients with relapsed or refractory diffuse large B-cell lymphoma (8). In their cases, thyroid abnormalities consisted of elevated thyroid autoantibodies and ultrasonographic thyroid inflammation, while thyroid function remained within the normal range and clinically apparent CRS was not described. In contrast, our cases developed biochemical thyrotoxicosis compatible with destructive thyroiditis after clinically significant CRS. These differences suggest that post-CAR T-cell thyroid abnormalities may include multiple phenotypes and warrant further investigation.

This study has several limitations. First, it was conducted at a single center and included only two cases of destructive thyroiditis, precluding conclusions regarding causality or risk factors. Second, surveillance was heterogeneous: thyroid function testing in the index case was symptom-triggered, whereas scheduled day 7 and day 14 monitoring was implemented in the subsequent 94 consecutive patients. This difference may have introduced surveillance and ascertainment bias. Third, baseline thyroid function and thyroid autoantibody status were determined from retrospective medical record review and were not measured uniformly immediately before CAR T-cell infusion; therefore, pre-existing subclinical thyroid dysfunction cannot be completely excluded. Fourth, systematic monitoring was limited primarily to days 7 and 14, and transient abnormalities outside this window or delayed thyroid dysfunction may have been missed. Fifth, minor biochemical abnormalities were common during the acute post-infusion period and may have reflected non-thyroidal illness, glucocorticoid administration, pre-existing thyroid disease, or other treatment-related factors. Sixth, thyroid scintigraphy was performed in only one of the two cases. Finally, in the patient with multiple myeloma, total T4 was not measured and FT4 was not confirmed using an alternative assay platform; therefore, assay interference cannot be completely excluded despite the low paraprotein burden and the clinical and scintigraphic findings supporting destructive thyroiditis. Our findings should be considered hypothesis-generating.

In conclusion, destructive thyroiditis may occur after CAR T-cell therapy and may be overlooked because its clinical manifestations overlap with CRS recovery and other post-CAR T-cell complications. The occurrence of both cases after grade ≥2 CRS represents a possible association but does not establish CRS severity as a risk factor. Thyroid function testing may be considered when otherwise unexplained tachycardia or persistent systemic symptoms occur after CAR T-cell therapy. Larger prospective studies with uniform baseline and longitudinal thyroid assessment are needed to determine the incidence, clinical course, risk factors, and optimal monitoring strategy.

## Data Availability

The data supporting the conclusions of this article are included within the article. Additional de-identified data are available from the corresponding author upon reasonable request, in accordance with applicable ethical and privacy requirements.
